# Quantitative Portal Vein Velocity of Liver Cancer Patients with Transcatheter Arterial Chemoembolization on Angiography

**DOI:** 10.1100/2012/830531

**Published:** 2012-07-31

**Authors:** Yung-Jen Ho, Mu-Bai Chang, Yang-Hsien Lin, Chun-Hsu Yao, Tzung-Chi Huang

**Affiliations:** ^1^Department of Biomedical Imaging and Radiological Science, China Medical University, 91 Hsueh-Shih Road, Taichung 404, Taiwan; ^2^Radiology Department, China Medical University Hospital, Taichung 404, Taiwan; ^3^Department of Biomedical Imaging and Radiological Sciences, National Yang-Ming University, Taipei 112, Taiwan

## Abstract

*Objective*. We applied optical flow method (OFM) to quantify relative velocities of blood flow using digital subtraction angiography (DSA) in the vascular analysis of hepatocellular carcinoma (HCC) patients who underwent transarterial chemoembolization (TACE) treatment. *Methods*. A total of 40 HCC patients treated by TACE were analyzed in this study. DSA imaging with a 12-inch field of view, 1024 × 1024 pixels and 4 frames/second was acquired. OFM developed for motion estimation is applied for blood flow estimation. Two acrylic phantoms were built to validate the method. *Results*. The relationship between the OFM and Doppler measurements was found linear with *R* ^2^ = 0.99 for both straight and curved tube phantoms. Quantitative blood flow distribution images of the portal vein region were presented. After TACE, the minimum, maximum and mean velocities in the portal vein all decreased (*P* < 0.05). Additionally, the velocity in the portal vein is significantly lower with a higher Child-Pugh score (*P* < 0.01). *Conclusions*. The present technique provides add-on quantitative information of flows to DSA and the hemodynamic analysis in relative quantifications of blood flow in portal vein of hepatocellular carcinoma patients using DSA.

## 1. Introduction

Transarterial chemoembolization (TACE) has become the first choice of interventional radiology treatments for hepatocellular carcinoma (HCC). Because of embolization of the artery, the reduction in the arterial blood supply to the tumor causes hypoxia and cell death to the tumor but usually spares the adjacent normal liver cells [1–3]. The chemotherapeutic materials for embolization include gelfoam, lipiodol, and cytotoxic agents [4]. TACE has been shown to reduce systemic toxicity and increase local control thus it is an effective treatment method which improves the therapeutic results [5, 6].

Hemodynamic model of vascular lesions in HCC region is essential for correct diagnosis and treatment strategy. Non-invasive imaging modalities for the modeling include ultrasound image, Doppler scan, magnetic resonance angiography (MRA), and CT angiography (CTA) [7–10]. However, the traditional ultrasound imaging usually cannot provide a detailed blood flow distribution because of its disadvantages of low spatial resolution, large signal attenuation through bones, and dependence on individual operator's skill and judgment. The measurement data could be different with different angle or machine. Although the Doppler ultrasound technique is able to obtain the blood flow velocity, the measurement using this technique can only provide such information for a single point. It cannot give a complete set of data for a large region to build the hemodynamic model. The key techniques used in MRA to evaluate the blood flow velocity include phase contrast (PC) and time of flight (TOF). These techniques with noninvasive, nonradiation and high in resolution are effective in the screening of vascular lesions, but the blood flow direction cannot be determined using these techniques [11]. The CTA can provide 2D and 3D images with high resolution, which presents the relationship between the lesion and the surrounding blood vessels, however hemodynamic information is excluded. 

Currently, the digital subtraction angiography (DSA) is the most commonly used technique in diagnosis, image guidance, or interventional treatment of vascular lesions. However, the quantitative information provided by this technique is very limited. Because of this, many papers discussed the methods of blood flow velocity measurement with angiography and compared the methods in principle, characteristics, advantage/disadvantage, and so forth. In blood flow velocity measurement, the vessel shape, pulse rate, the heterogeneity in velocity, and multidirectional nature make the measurement complicated [12]. Recently, Siemens released the syngo iFlow imaging that allows for the dynamic flow evaluation with the visualization in full color by optical flow method, which potentiates DSA with higher temporal resolution. Shpilfoygel et al. reviewed over 100 manuscripts related to flow measurement with DSA, and illustrated the advantage of using optical flow-type methods [13]. Additionally, a previous study pointed out that the blood flow estimation using optical flow method is close to the real values [14].

Currently, several imaging modalities have been developed to determine the diagnosis of HCC, none has emerged with the hemodynamic information with high resolution inside whole vessels. In this study, we applied the optical flow method to quantify relative velocities of blood flow using angiography and investigated the vascular analysis on HCC patients who underwent TACE treatment. The blood flow velocity was calculated by applying the optical flow method on the images taken for the TACE treatment. The calculated velocity was compared with the Doppler ultrasound measurement. The velocity in the portal vein was compared between before and after TACE. The relationship between the Child-Pugh score and the flow velocity in the liver portal vein was analyzed. 

## 2. Materials and Methods 

### 2.1. Patients and Angiography

A total of 40 TACE cases treated in the Radiology Department, China Medical University Hospital, were analyzed in this study. (1) Among them, the information collected from 27 patients included the DSA images, velocity measurements by Doppler ultrasound, and the patients' liver function and other related pathological data. For an accurate flow velocity evaluation, every patient of these 27 cases had DSA and Doppler ultrasound imaging before and after TACE. (2) All 40 cases were involved in the Child-Pugh score analysis, of which 32 cases were with score A and 8 with score B. The age of the patients ranged from 55 to 74 years old with 26 male and 14 female. The average flow velocities at the front, middle and back sections in the portal vein were measured using the Doppler ultrasound technique and compared with the calculated values. To avoid differences introduced by different operators, all measurements were performed by the same radiologist. This study was approved by the China Medical University Hospital, Taiwan (DMR100-IRB-181).

 The DSA imaging used PHILIPS Multi Diagnost Eleva with a 12-inch field of view and 1024 × 1024 pixels. The tube voltage was in the range of 40–150 kV and the tube current 10–1000 mA. The temporal resolution was 4 frames/second. Two contrast injection methods were used in the DSA imaging: superior mesenteric artery (SMA) injection to observe the blood flow from SMA to superior mesenteric vein (SMV) and celiac axis injection to get the blood flow information from the spleen vein returning to the portal vein. The former method was used in blood flow analysis around the portal vein, and the latter was to evaluate the blood flow distribution after TACE. 

### 2.2. Transarterial Chemoembolization (TACE)

Chemoembolization generates embolization with the intra-arterial administration of drugs directly to the tumor. During arterial injection, the lipiodol/chemotherapeutic solution is delivered preferentially to the tumor due to the blood flow characteristics [4, 15]. After disinfection, the doctor punctures the femoral artery using a puncture needle and put a catheter in there. Normal saline and 1 cc of heparin is injected to prevent solidification of blood in the catheter. The catheter is then one-way locked to prevent blood spray and the contrast is injected at SMA to make sure the location is correct. Alprostadil (Prostaglandin E1, PGE1) is then injected to expand the blood vessel and the automatic injector is installed. The blood to the hepatic artery flows from the celiac axis. When the contrast is injected to the celiac axis, the location of the lesion can be determined. With the lesion location determined, the oily contrast Lipiodol, which intends to cover the lesion, is injected to the entrance artery section closest to the lesion site with 20 mg of chemodrug Adriamycin and 1 cc of cefa mixed with Gelfoam powder. Embolization is then performed in the artery leading to the tumor site, which stops nutrition to the tumor cells and eventually kills the tumor cells.

### 2.3. Blood Flow Estimation

Mutual information was used for initial image registration due to organ motion resulted from heartbeat, respiration, and intestinal peristalsis during the image acquisition. A series of successive images were aligned together based on maximization of mutual information as the preprocessing for blood flow estimation [16–18]. Optical flow method (OFM) developed for motion estimation is applied on the aligned images for blood flow estimation in this study [19]. The spatial accuracy of blood flow estimation calculated by OFM was reported in the previous studies [20–23]. In this study, the OFM algorithm was applied to calculate the velocity of blood flow on two successive images of DSA. The velocity matrix includes lateral and inferior-superior displacements, respectively, for each pixel in the images. The OFM calculation equation is implemented in the computer program as shown below:

(1) ν(n+1)=ν(n)+∇f(∇f·ν(n)+∂f/∂tα2+||∇f||2),

where *n* is the number of iterations and *ν* ^(*n*)^ is the average velocity derived from the surrounding voxels, *f* is the image intensity, and *α* is the weighting factor of which the value is empirically set at 5 for DSA image.

### 2.4. Phantom Study

**Figure 1:**
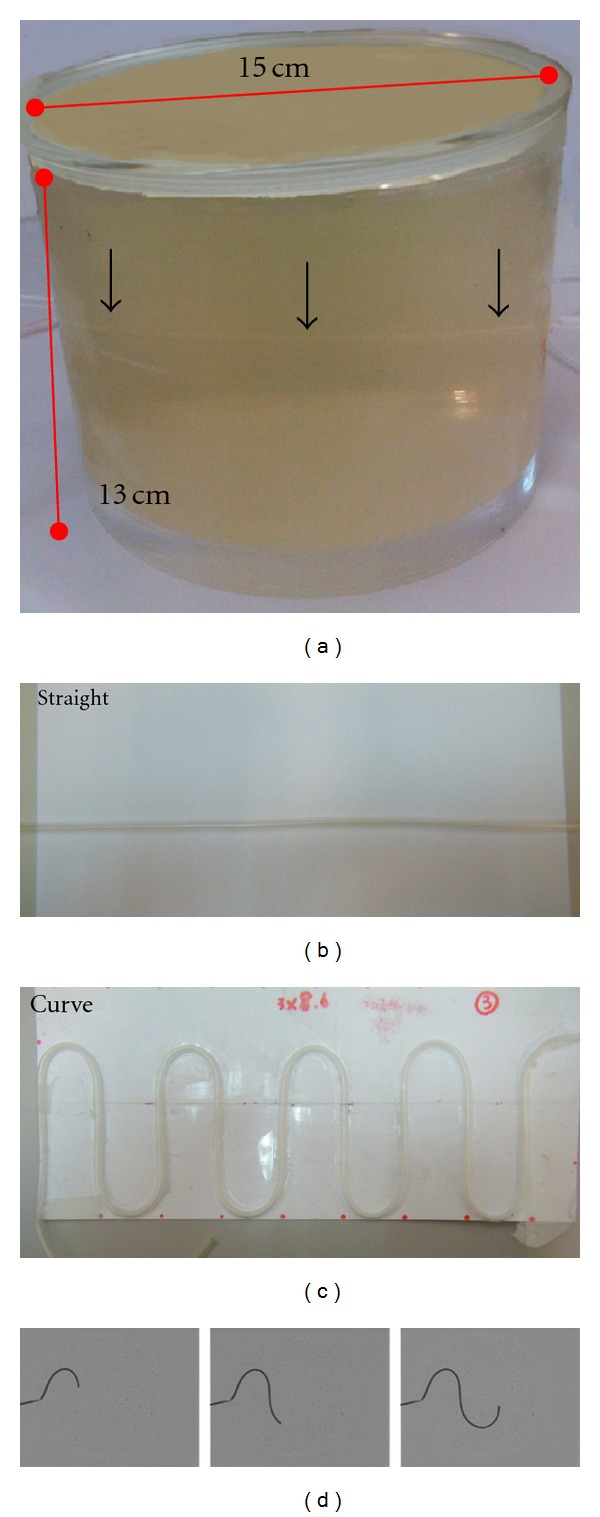
Phantom (a) filled with gelatin; (b) plastic tubes—straight; (c) Plastic tubes—curve; (d) an example image.

For the feasibility study using OFM for blood flow velocity calculation, two 15 × 15 × 13 cm^3^ acrylic phantoms (Figure 1(a)) were designed and made to get the accuracy of this method, one with straight tube (Figure 1(b)) and the other with curved tube (Figure 1(c)). The phantoms were filled with gelatin (porcine skin, type A, 300 Bloom, Sigma Aldrich) for ultrasound imaging. The diameter of the tubes was 4.0 mm. A pump was connected to the tube to inject the diluted contrast through the phantom when imaging using Doppler ultrasound (Siemens Acuson X150). A CH5-2 convex probe for abdomen diagnosis was used. The frequency was 2–5 MHz and the Doppler angle was 60 degrees. A total of 13 points were selected for the straight tube phantom to scan for the average flow velocity. For the curved tube phantom, 11 points were selected. The DSA imaging was done at the same time using Philips Multi Diagnost Eleva with a temporal resolution of 4 frame/s (Figure 1(d)). OFM was used to calculate the flow velocity and the values were compared with the Doppler ultrasound measurements. 

## 3. Results

### 3.1. Accuracy in Phantom

For the straight tube phantom, the average velocity by the OFM was 75.36 pixel/frame, while the average flow velocity over the 13 points by Doppler ultrasound imaging was 69.44 cm/s. For the curved tube phantom, the average velocity by OFM was 79.4 pixel/frame, and the average velocity by Doppler ultrasound was 66.23 cm/s. The relationship between the OFM calculations and Doppler ultrasound measurements was found to be linear with *R* ^2^ = 0.99 in linear data fitting for both straight and curved tube phantom. 

### 3.2. Clinical Patients Analysis

**Figure 2:**
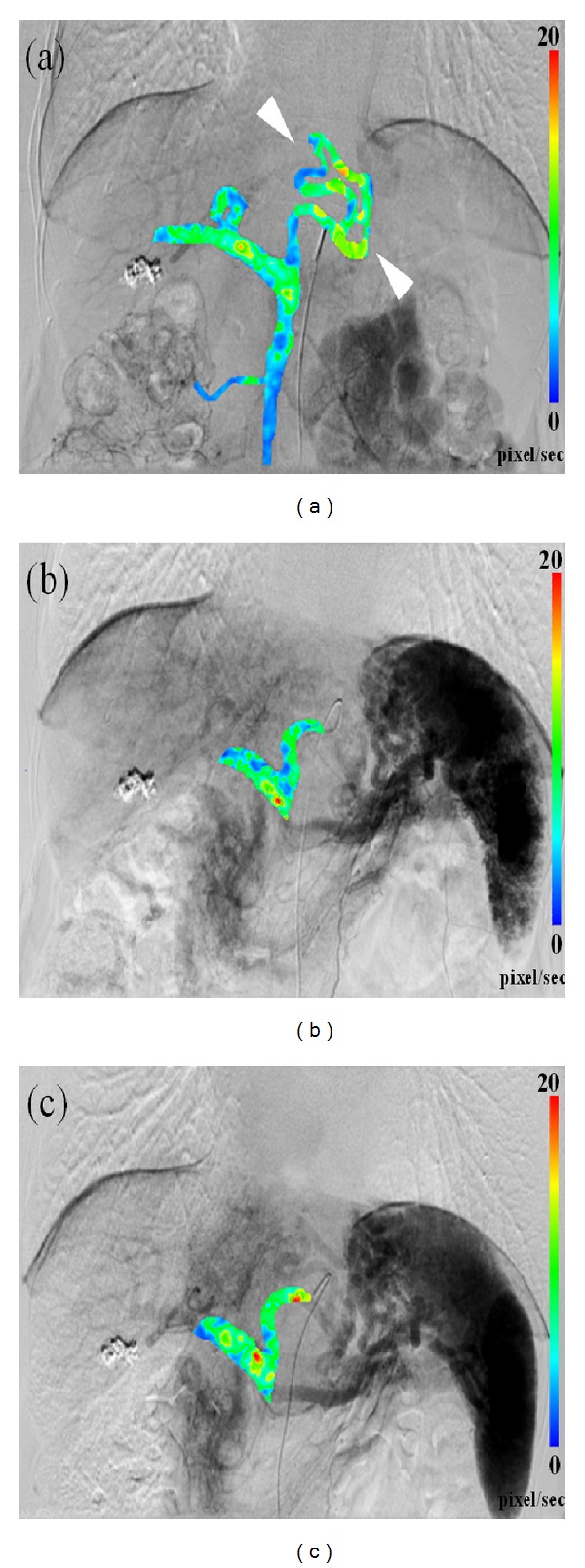
An example of the blood flow distribution with color coding. (a) The portal vein flow image with the contrast injected in SMA. (b) The portal vein flow image before TACE with the contrast injected in celiac axis. (c) The portal vein flow image after TACE with the contrast injected in celiac axis.

For clinical patients data relationship between the OFM calculated blood velocity values and the Doppler ultrasound measured ones over the 27 TACE cases, the fitted equation was *y* = 24.65 × −2.33 with *R* ^2^ = 0.69 and the correlation coefficient was 0.83 which means that the data were highly positively correlated. The blood flow distribution image uses color coding to illustrate the spatial blood flow velocity variation with red meaning fast flow.  Figure 2 is a set of blood flow mapping images of the portal vein region for an 80 years old female patient. Because of the high pressure in the portal vein, this patient had varicose portal vein in the left lobe of liver. In this figure, the flow velocity values were normalized for comparison.  Figure 2(a) shows the portal vein circulation with the contrast injected in SMA. The varicosis can be clearly seen. Figures 2(b) and 2(c) show the blood flow from the spleen vein to the portal vein before and after the embolization, with contrast injected in the celiac axis. The blood flow changes before and after the treatment can be observed in this figure. The dynamic flows mapping with color-coding superimposed on conventional DSA before and after the embolization were presented in supplementary material. 

**Figure 3:**
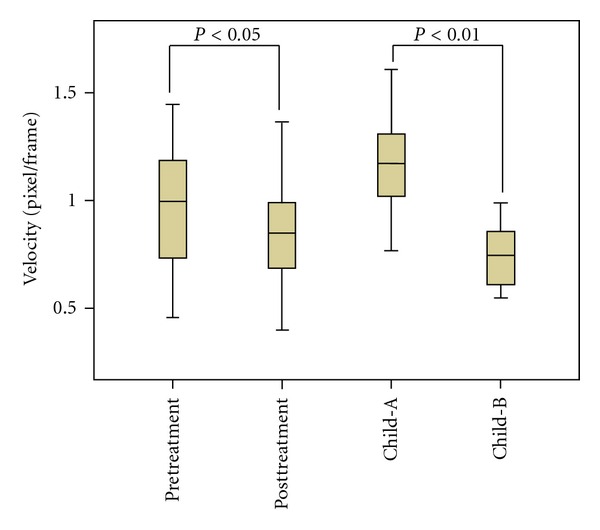
The statistic comparison of blood flow before and after TACE/Child-Pugh A-B.

Figure 3 shows the comparison of the flow velocity before and after TACE treatment/ Child-Pugh A/Child-Pugh B for all the 40 cases. It indicates that after TACE, the minimum, maximum and mean velocities in the portal vein all decreased (*P* < 0.05). Flow in portal vein with Child-Pugh A and B were 1.18 ± 0.20 and 0.74 ± 0.16 pixel/frame, respectively. Additionally, as the Child-Pugh score is an indicator of the degree of the cirrhosis of the liver, with a higher score, the velocity in the portal vein is significantly lower (*P* < 0.01).

## 4. Discussion

With quantitative flow estimation, DSA provides not only high spatial and temporal resolution of images, but also the hemodynamic information. The relative quantitative blood flow estimation by applying optical flow method to perioperatively monitor TACE patient receiving embolization was presented in this study. Color coding superimposed on DSA image quantitatively illustrates the flow value determined by OFM, for example, the red color (on image and color bar of Figure 2) quantitatively indicates the flow of 20 pixel/second. However, on a conventional angiography image, the hemodynamics is illustrated on sequential opacification of the vascular structures with grey scales. The interpretation is qualitatively rather than quantitatively, and usually based on physicians experience and observation. Optical flow analysis enhanced the inherent superior temporal value of DSA, which transformed it into a powerful parametric hemodynamic markerfor therapeutic implication.

**Figure 4:**
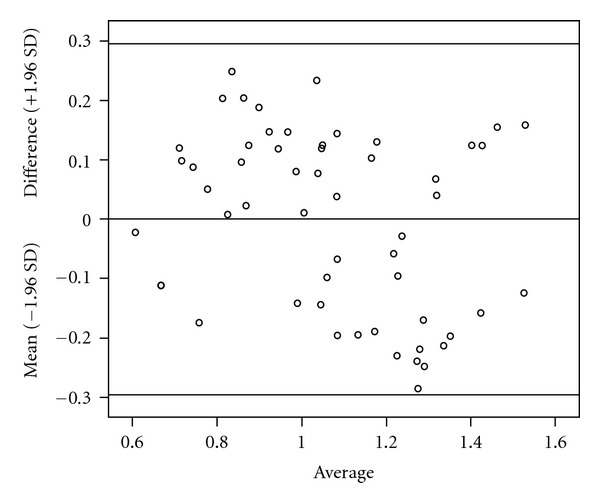
Bland-Altman difference plot by OFM and Doppler ultrasound measurement.

OFM basically calculates the flow information according to the changes in image intensity on two successive images and the unit of blood flow was pixel per second. Flow motion defined by pixel change versus time frame was different from actual velocity generally used in distance with time (cm/s). In the phantom experiment, the flow velocity was measured by Doppler ultrasound and the DSA images were analyzed and the flow velocity was calculated using OFM. The relationship between pixel/frame and cm/second is strongly associated with each other (*R* ^2^ = 0.99). This indicates that applying OFM on DSA images to get the blood flow velocity distribution is feasible and practical. The concept of Bland-Altman difference plot is to calculate the difference of a physical quantity using two different methods and plot the difference distribution. If the difference points are around 0, then one can conclude that the two methods are close to each other. The horizontal axis in Figure 4 is the average velocity by OFM and Doppler ultrasound, the vertical axis is their difference. The horizontal line in the middle is the average difference and the upper and lower lines define the 95% confidence region (average ± 1.96 SD). All the difference points are within the 95% confidence region, which means the OFM calculated and the Doppler ultrasound measured velocity values are all within the tolerant error range, and OFM calculated velocity values are consistent with the measurements. OFM was used in this study to calculate the spatial motion of the contrast on the DSA images with a sampling rate of 4 frames/s. The blood velocity was thus calculated using the spatial displacement and the time interval. There have been some reports of using OFM to calculate blood flow. In 1995, Imbert published a paper on the usage of OFM in blood flow calculation on DSA images of simulated blood vessels [24]. The correlation coefficient between the calculations and the real values was reported to be 0.99 and the errors were within 1.5%. Based on the papers, the accuracy of using OFM to calculate blood flow velocity is high. However, the majority of the papers were based on the experiments in which phantoms were used to simulate blood flows. The present study not only applied OFM in a phantom study but also used OFM on clinical DSA images in blood flow velocity calculations.

The analysis of the blood velocity in the portal vein versus the Child-Pugh score was aimed to get the relationship between the cirrhosis of the liver and the velocity in the portal vein. Clinically, the Child-Pugh score is used to evaluate the degree of the cirrhosis of the liver, higher score means more serious of the cirrhosis. Our analysis demonstrated that the velocity in the portal vein is lower with higher Child-Pugh score (Child-Pugh A: 1.18 ± 0.20 pixel/frame, Child-Pugh B:  0.74 ± 0.16 pixel/frame), which is consistent with the 1991 report by Zironi et al. [25] in which the velocity in the portal vein versus the liver portal vein pressure was reported. Because one of the common complications of the cirrhosis is high liver portal vein pressure, with more serious cirrhosis, the probability of high liver portal vein pressure is higher, thus the velocity in the portal vein is lower. Receiver operator characteristic curve was applied to determinate of the Child-Pugh A-B score versus the velocity in the portal vein [26]. The horizontal axis is the specificity and the vertical axis is the sensitivity. The specificity represents the ratio of the correctly given the Child-A score with a certain mean velocity in the portal vein while the sensitivity represents the ratio of the correctly given the Child-B score based on the mean velocity in the portal vein. The curve demonstrates that the trend is (0, 1), indicating that the blood velocities in the portal vein can clearly distinguish the Child-Pugh scores. The area under the curve is 0.9688, very close to 1, which is an indication that the accuracy of using the velocity in the portal vein to determine Child-Pugh score is high.  Table 1 lists the sensitivity, specificity, and accuracy of using the velocity in the portal vein to determine the Child-Pugh score A or B of the top 6 cases. The accuracy was higher than 80% for all the cases with a maximum value of 92.5%.

### 4.1. Limitation of the Method

OFM is an image intensity gradient based deformable registration method. It detects the grey level changes between images taken at different time and determines the pixel-to-pixel correspondence between the images. Two fundamental assumptions are involved in OFM registration: (1) the intensity of a certain point in an image does not change with time and (2) the surrounding points move with a similar manner, which is called the smoothness of motion assumption. In this study, OFM was applied on real clinical DSA images. The following limitations must be considered: (1) because of the smoothness of motion assumption, the blood flow motion must not violate this assumption. (2) Since OFM looks for the displacement of the corresponding points on two images and calculates the velocity based on the displacement values, the result is relative velocity but not absolute velocity. Considering these two issues, the portal vein was selected for the velocity calculation. In addition to the consideration that the portal vein is an important vessel in the liver, the blood flow in the portal vein matches the smoothness of motion assumption for OFM's application was the other reason this site was selected. Additionally, before the statistical analysis on the calculated velocity values, the values were normalized and then compared with the Doppler ultrasound measurements. This normalization process makes the statistical analysis meaningful. 

## 5. Conclusions

The imaging technique introduced in this paper provides add-on quantitative information of flows to DSA. It helps the conversion of the qualitative hemodynamic information on DSA, that is usually based on physicians experience and observation, into objective and parametric information and can subsequently help refine clinical therapeutic strategy. The presented study is the first report of hemodynamic analysis in relative quantifications of blood flow in portal vein of HCC patients using DSA. DSA from angiography with quantitative blood flow information may assist analysis in the treatment of TACE. 

## Supplementary Material

The dynamic flows mapping with color-coding superimposed on conventional DSA before and after the embolization.

## Figures and Tables

**Table 1:** List of top six value of mean potion vein flow for sensitivity and specificity in detection of Child-Pugh score A to B.

*V*_mean_ (pixel/frame)	Sensitivity	Specificity	Accuracy
0.879829	88%	97%	92.5%
0.988918	100%	84%	87.5%
0.914252	88%	94%	92.5%
0.996215	100%	81%	85%
0.964224	88%	91%	87.5%
1.004337	100%	78%	82.5%
