# Supplementary material for: Quantitative Portal Vein Velocity of Liver Cancer Patients with Transcatheter Arterial Chemoembolization on Angiography
Source: ScientificWorldJournal. 2012 Jul 31;2012:830531. doi: 10.1100/2012/830531 (PMC3417180; doi:10.1100/2012/830531)

■ Liver Angiography

Before TACE- Celiac Trunk

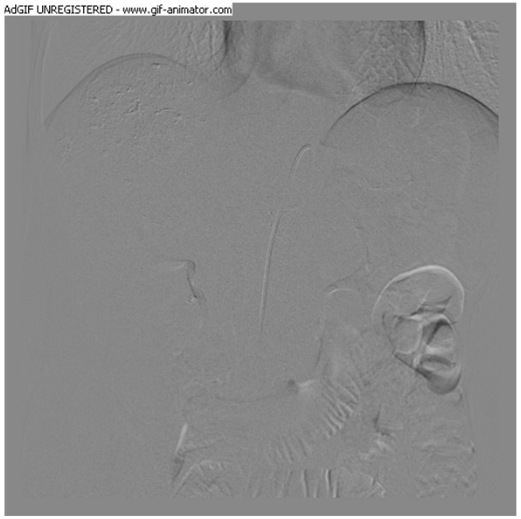

After TACE- Celiac trunk

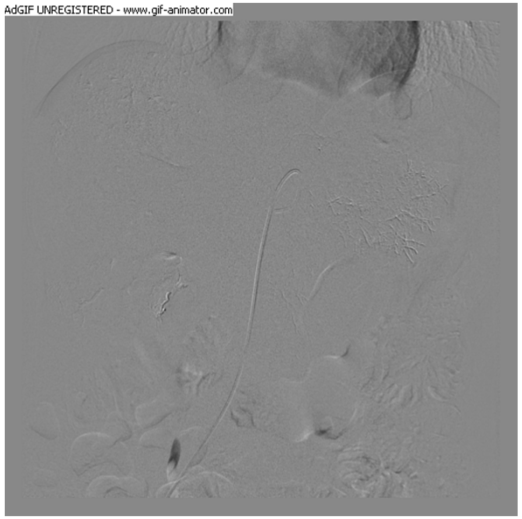

■ Quantification in flow information

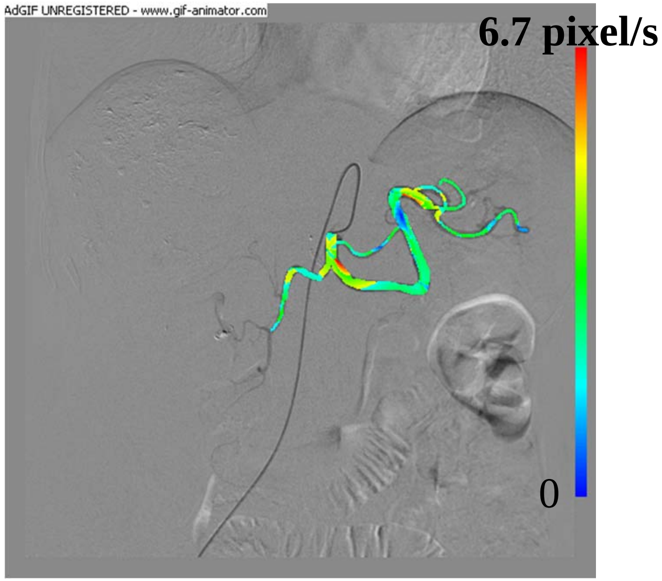

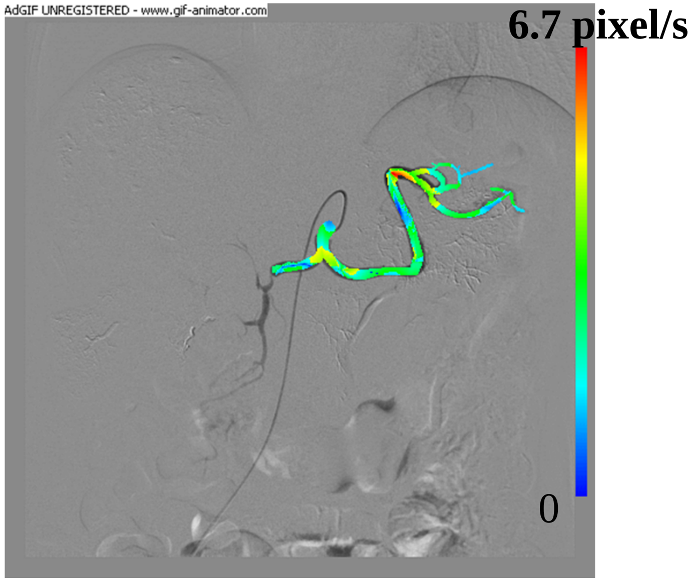

Supplement: Supplementary file 1 — The dynamic flows mapping with color-coding superimposed on conventional DSA before and after the embolization. [file 830531.f1.pdf]
